# Zika virus tropism and interactions in myelinating neural cell cultures: CNS cells and myelin are preferentially affected

**DOI:** 10.1186/s40478-017-0450-8

**Published:** 2017-06-23

**Authors:** Stephanie L. Cumberworth, Jennifer A. Barrie, Madeleine E. Cunningham, Daniely Paulino Gomes de Figueiredo, Verena Schultz, Adrian J. Wilder-Smith, Benjamin Brennan, Lindomar J. Pena, Rafael Freitas de Oliveira França, Christopher Linington, Susan C. Barnett, Hugh J. Willison, Alain Kohl, Julia M. Edgar

**Affiliations:** 1 MRC-University of Glasgow Centre for Virus Research, G61 1QH, Glasgow, Scotland UK; 2 Institute of Infection, Immunity and Inflammation, College of Medical Veterinary and Life Sciences, University of Glasgow, G12 8TA, Glasgow, Scotland UK; 3 Oswaldo Cruz Foundation/Aggeu Magalhães Institute, Department of Virology, UFPE Campus-Cidade Universitária, Recife/PE, Brazil; 4 Department of Neurogenetics, Max Planck Institute for Experimental Medicine, Hermann-Rein-Strasse 3, 37075 Goettingen, Germany

**Keywords:** Zika virus, Tropism, Murine, Central nervous system, Peripheral nervous system, Myelin, Axon

## Abstract

The recent global outbreak of Zika virus (ZIKV) infection has been linked to severe neurological disorders affecting the peripheral and central nervous systems (PNS and CNS, respectively). The pathobiology underlying these diverse clinical phenotypes are the subject of intense research; however, even the principal neural cell types vulnerable to productive Zika infection remain poorly characterised. Here we used CNS and PNS myelinating cultures from wild type and *Ifnar1* knockout mice to examine neuronal and glial tropism and short-term consequences of direct infection with a Brazilian variant of ZIKV. Cell cultures were infected pre- or post-myelination for various intervals, then stained with cell-type and ZIKV-specific antibodies. In bypassing systemic immunity using ex vivo culture, and the type I interferon response in *Ifnar1* deficient cells, we were able to evaluate the intrinsic infectivity of neural cells. Through systematic quantification of ZIKV infected cells in myelinating cultures, we found that ZIKV infection is enhanced in the absence of the type I interferon responses and that CNS cells are considerably more susceptible to infection than PNS cells. In particular, we demonstrate that CNS axons and myelinating oligodendrocytes are especially vulnerable to injury. These results have implications for understanding the pathobiology of neurological symptoms associated with ZIKV infection. Furthermore, we provide a quantifiable ex vivo infection model that can be used for fundamental and therapeutic studies on viral neuroinvasion and its consequences.

**Electronic supplementary material:**

The online version of this article (doi:10.1186/s40478-017-0450-8) contains supplementary material, which is available to authorized users.

## Introduction

Zika virus (ZIKV; family *Flaviviridae*, genus *Flavivirus*) is a positive stranded, mosquito-borne RNA virus associated with the peripheral neuropathy, Guillain-Barré syndrome (GBS), as well as microcephaly and other congenital brain abnormalities [5, 6, 40, 95, 96]; the impact on embryos and subsequent developmental problems are now referred to as Zika congenital syndrome (for example see [51, 58]). The risk of microcephaly is highest if mothers are infected during the first trimester of pregnancy ([86] and references within) but it is likely other neurological phenotypes arise following infection at later developmental stages. How ZIKV causes GBS, classically triggered through autoimmune responses against bacterial and viral infections [22, 31], remains to be explored and the interplay between direct infection and para- or post-infectious autoimmunity needs clarification [90].

ZIKV is closely related to other mosquito-borne flaviviruses such as dengue (DENV), Japanese encephalitis (JEV), St. Louis Encephalitis (SLEV) and West Nile (WNV) viruses [29]. Originally isolated in Africa, the virus has emerged in many regions of the world that can sustain transmission by competent vectors. In the current outbreak in the Americas, ZIKV is believed to be transmitted mainly by *Aedes aegypti* mosquitoes [25].

A number of arthropod-borne flaviviruses are neurotropic, including mosquito-borne JEV, WNV and SLEV, but also tick-transmitted flaviviruses such as tick-borne encephalitis virus (TBEV) [71]. DENV is not generally considered neurotropic but has also been associated with neurologic disease [78]. Of the mosquito-borne flaviviruses, the neurological complications arising from JEV and WNV infection are documented best. JEV, an important pathogen across Asia, is associated with meningitis and encephalitis [54, 79], whilst WNV is linked with encephalitis, particularly in the elderly [56]; with long term neurological sequelae in convalescent patients [97].

At present, the determinants underlying ZIKV viral tropism (both host and viral) are unclear, although several hypotheses have been discussed [2]. Importantly, ZIKV has been shown to replicate in human placental and foetal cells [21], and virus has been found in human foetal tissues [18, 51, 55, 67, 73]. Studies in primates reproduce some of the effects seen in human infection, including brain lesions, confirming a causal link between ZIKV infection and neurological outcomes [1, 43]. Experimental studies on the neurotropism of ZIKV demonstrate it can infect human neural cell-derived organoid systems/neurospheres, neuroepithelial/neural stem cells and radial glia [15, 26–28, 49, 64, 68]; variations in infection patterns and host responses have been attributed to differences between ZIKV strains [26, 75, 99]. Whilst there are few data on the neuropathogenesis of ZIKV infection, infected human-derived neural crest cells produce cytokines at levels that kill or cause aberrant differentiation of neural progenitors [4], and expression of genes involved in cell cycle and neural differentiation are altered in ZIKV-infected human iPS-cell derived neurospheres [28].

Mouse models have been used to study placental damage, infection of foetuses, testicular infection, neuropathogenesis, antibody protection and ZIKV strain specific effects [14, 24, 32, 41, 47, 52, 53, 72, 76, 80, 87]. Whilst animal models are undoubtedly important, cell culture systems (i) facilitate manipulation of experimental conditions, (ii) yield relatively rapid results and (iii) inform animal studies, thus refining and reducing the use of experimental animals. Here we infected CNS and PNS ‘myelinating’ cultures derived from embryonic wild type and type I interferon incompetent mice with a Brazilian, patient-derived isolate of ZIKV, to define neural tropism and short-term consequences of direct infection. Myelinating cultures, which replicate several aspects of the intact nervous systems, including complex cell-cell interactions, were infected pre- and post-myelination, mimicking late foetal and early postnatal life. We found that all major CNS cell types were susceptible to productive infection in type I interferon incompetent cultures and CNS axons and myelinating oligodendrocytes were particularly vulnerable to injury; an observation that might be important for understanding the less well-characterised neurological phenotypes in both microcephalic and non-microcephalic cases. In contrast, PNS infection rates were generally very low, even in absence of type I interferon responses, suggesting that GBS is unlikely the result of direct viral infection of the PNS.

## Material and methods

### Mouse breeding and genotyping

*Ifnar1* knockout (KO; type I interferon incompetent) and wild type (WT) mice on a 129S7/SvEvBrdBkl-Hprtb-m2 background (B&K Universal) were maintained in Tecniplast 1284 L Blue line IVC cages, in a 12 h light/dark cycle and provided ad libitum with sterile food and water. Mice were time-mated and pregnant females were killed by CO_2_ overdose on embryonic day (E) 13. All animal studies were approved by the Ethical Committee of the University of Glasgow and licensed by the UK Home Office (Project Licence number PPL 60/4363). Genomic DNA was extracted from ear biopsies using a protocol modified from [88]. Briefly, ear notches were heated to 95 °C for 90 min in 50 mM NaOH. Following neutralisation with 10% *v*/v 1 M Tris pH 5, the resultant solution was vortexed to release DNA and 1 μl was used for PCR.

### Genotyping

For PCR, RedTaq polymerase (Sigma Aldrich) was used. Briefly, each reaction contained 1× reaction buffer including 0.2 mM dNTPs, 0.2 μM primer, 0.05 U/μl polymerase and 1 μl ear biopsy lysate. An initial heating step of 95 °C for 2 min was followed by 35 cycles of 95 °C 1 min, 60 °C 1 min and 72 °C 2 min. For completion of syntheses, samples underwent a final step of 72 °C for 5 min before being cooled to 4 °C. The products were analyzed by agarose gel electrophoreses according to standard protocols.

### Cell culture

CNS myelinating cultures were established as described previously [82, 83], with minor modifications. Briefly, E13 (day of plug E0) mouse spinal cords were isolated and stripped of their meninges then dissociated into a single cell suspension using trypsin and trituration through a glass pipette. Cells were plated at 150,000 cells per 13 mm diameter glass coverslips coated with poly-L-lysine (0.1 mg/ml in boric acid buffer pH 8.4); coverslips were located in 3 s inside 35 mm Petri dishes. Cells were plated initially in 12.5% horse serum, which was gradually withdrawn through feeding every 2nd or 3rd day with serum-free differentiation medium (DMEM [4.5 mg/ml glucose], 100 U/ml penicillin, 100 μg/ml streptomycin, 10 ng/ml biotin, 1% N1, 50 nM hydrocortisone, and 10 μg/ml insulin; the last for the first 12 days only. All reagents were from Sigma-Aldrich, Dorset, UK. Cells were maintained in 5% C0_2_ at 37 °C. PNS myelinating cultures were established as described previously [66], with modifications. Briefly, whole dorsal root ganglia (DRG) were plucked from the E13 mouse spinal cord meninges with fine forceps and plated singly onto Matrigel (1:3 in EMEM)/poly-D-lysine (0.1 mg/ml) coated 13 mm diameter coverslips in 80 μl growth media (MEM [4 mg/ml glucose], 100 U/ml penicillin, 100 μg/ml streptomycin, 10% horse serum, 50 ng/ml nerve growth factor). DRGs were cultured overnight before a further 400 μl growth medium was added. They were maintained throughout in 5% CO_2_ at 37 °C. At day in vitro ﻿﻿﻿(DIV) 8, growth medium was replaced with myelinating medium (MEM [4 mg/ml glucose], 100 U/ml penicillin, 100 μg/ml streptomycin, 5% horse serum, 50 ng/ml nerve growth factor, 1% N2, 20 μg/ml bovine pituitary extract, 0.5 μM forskolin, 50 μg/ml ascorbic acid) and 50% was exchanged with fresh medium every 2–3 days. Reagents were from Sigma-Aldrich, Dorset, UK or Invitrogen, Paisley, UK.

### Infection of cultures with ZIKV

The low passage Brazilian strain of ZIKV, *ZIKV/H*. *sapiens/Brazil/PE243/2015* (GenBank accession number KX197192; abbreviated ZIKV PE243; in this paper referred to only as ZIKV) was used; its origin and history have been previously described [17]. Both CNS and PNS cultures were transported between geographically separated sites at room temperature in a sealed container containing 5% CO_2_ and allowed to equilibrate at 37 °C in 5% CO_2_ overnight. CNS/PNS cultures (minimum of 20 or 12 coverslips, respectively, per independent experiment) were infected with ZIKV at a multiplicity of infection (MOI) of 0.3 or 3.0 for 1 h at 37 °C in PBS supplemented with 2% foetal bovine serum (FBS). Controls (mock-infected; minimum of 5 CNS and 6 PNS coverslips per independent experiment) were treated in parallel with vehicle only (2% FBS in PBS). Following incubation, virus was aspirated and the cultures returned to serum-free differentiation medium (CNS) or myelination medium (PNS). At the indicated times post infection, cultures were fixed with 8% paraformaldehyde for 1 h at room temperature and subsequently stored in PBS at 4 °C for up to 2 weeks before staining. CNS/PNS cultures utilised in 6/12 days post infection (dpi) experiments were infected as described, and maintained with media replenishments every 2 days until fixation at 6/12 dpi before fixation, as described above.

### Test for anti-ZIKV proliferative effect of PNS culture media

To test if the media in which the DRG explants were maintained was inhibitory to ZIKV replication, A549 cells (a human cell line which we have previously characterised for ZIKV infection [17]) were infected with ZIKV at MOI 0.3 and maintained for 72 h post infection (hpi) in (i) DMEM GlutaMAX supplemented with either 10% horse serum (used to supplement PNS myelinating media), 10% FBS (used to maintain A549 cells) or (ii) PNS myelinating media supplemented with either 10% FBS or 10% horse serum. At 72 hpi cells were fixed with 8% paraformaldehyde and analysed by immunofluorescence using an antibody directed against the ZIKV envelope protein (clone 0302156 Aalto Bio; 1 in 500). Imaging was performed using an EVOS Fl microscope.

### Immunocytochemistry

Post-fixation, myelinating cultures were permeabilised in ethanol (−20 °C; 10 min) and incubated in primary antibodies in 10% goat serum overnight at 4 °C. Mouse anti-ZIKV [clone 0302156 Aalto Bio; 1 in 500] was used in combination with one or other of the following cell-type specific antibodies: rat anti-PLP/DM20 [Clone AA3, kindly provided by Dr. Steven Pfeiffer, Connecticut; 1 in 400]; rabbit anti-NeuN [Millipore; 1 in 750], rabbit anti-S100 [Dako; 1 in 400], rabbit anti-GFAP [Dako; 1 in 1000], rabbit anti-NG2 [Millipore; 1 in 200], rat anti-MBP [AbD serotec; 1 in 500], or rat anti-F480 [AbD serotec; 1in 600]). Rat anti-MBP was also used in combination with mouse SMI31 anti-phosphorylated heavy and medium chain neurofilament [Biolegend; 1 in 1500] and rat anti-F480 in combination with mouse SMI32 anti-non-phosphorylated neurofilament heavy chain [Sternberger; 1 in 1500] or mouse anti-β-Tubulin III [Sigma; 1 in 200]. After washing, secondary antibodies (goat anti-mouse IgG1 Alexa 488 and goat anti-rat IgG or goat anti-rabbit IgG or goat anti-mouse IgG2a Alexa 594; Invitrogen) were applied for 1 h at room temperature. Coverslips were mounted on glass slides in Citifluor mounting medium with DAPI (1 ng/ml; Electron Microscopy Sciences Pennsylvania US) and sealed with nail enamel.

### Image capture

For cell quantification, fluorescence microscopy and image capture were performed using an Olympus IX70 microscope with standard epifluorescence optics and Image Pro Plus 6 software. To avoid bias, fields of view (FoV) were selected in the blue (DAPI) channel then images were captured (10 images per coverslip) at ×20 magnification in the red (cell-type specific), green (ZIKV) and blue channels. Representative images for illustration were obtained using the Olympus IX70 microscope and Image Pro Plus 6 software; a Zeiss LSM 710 inverted confocal microscope and Zen Black software; or a Zeiss AxioImager Z1 with ApoTome structural illumination attachment and Zeiss Zen 2 software.

### Quantification of cells

Rectangular areas of interest (AoI) of 148,427 μm^2^ and 20,000 μm^2^ were placed on each image and immunostained cells or DAPI +ve nuclei, respectively, within and touching west and north borders were quantified. Only immunopositive structures with a DAPI +ve nucleus qualified as cells. The average cell density per AOI was converted to cells/mm^2^ using the formula: cell density per AOI/area of AOI μm^2^ × 1,000,000. Pyknotic nuclei were distinguished from healthy nuclei on the basis of size, and homogeneity and intensity of DAPI staining; pyknotic nuclei being condensed and intensely labelled.

### Quantification of CNS myelin

Anti-MBP labels mainly myelin and myelin-like sheaths whilst anti-PLP/DM20 labels myelin, myelin-like sheaths and oligodendrocyte cell bodies. Using digital images of representative fields of view of immunostained cultures, the area occupied by MBP or PLP/DM20 staining (pixels per AOI) was quantified using CellProfiler software. Pipelines are available at https://github.com/muecs/cp.

### Statistical analysis

Analyses were performed using Graphpad Prism4 software (GraphPad Software Inc., San Diego, CA). Significance is indicated as <0.05 (*), <0.01 (**) and <0.001 (***). A paired, one or two tailed Student’s t test was used to compare cell densities/myelin area between mock -infected and ZIKV-infected cultures, using *n* = 3 independent cultures i.e. cultures generated from 3 independent pregnant dams (2–4 coverslips per cell type and 12–20 coverslips per DAPI count were quantified and averaged for each independent culture).

## Results

### ZIKV infection is enhanced in the absence of type I responses

To determine the intrinsic infectivity of neural cells by a Brazilian strain of ZIKV (ZIKV PE243, see Materials and methods), we prepared myelinating (i) mixed spinal cord cell and (ii) dorsal root ganglia explant cultures to model the multi-cellular environment of the CNS and PNS, respectively. Cultures were prepared from WT and *Ifnar1* KO (type I interferon deficient) mouse embryos. *Ifnar1* KO cultures were indistinguishable from WT cultures with respect to cellular composition and myelination (Additional file 1: Figure S1a and b [CNS] and 1c and d [PNS]).

**Fig. 1:**
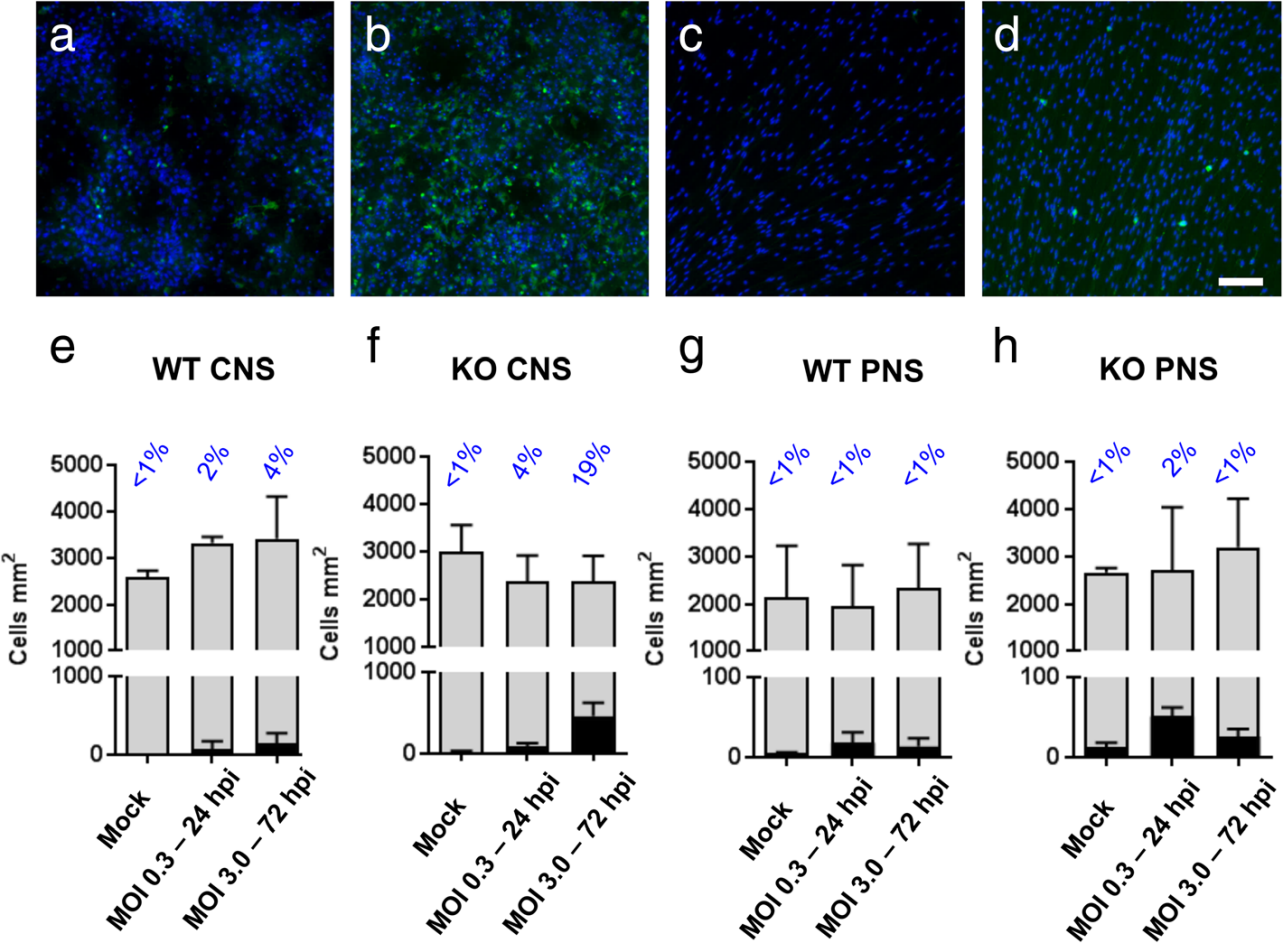
ZIKV infection is limited by the type I interferon response in neural cell cultures. **a-d** Micrographs of infected cells in myelinating cultures from wild type (WT) CNS (**a**), *Ifnar1* knockout (KO) CNS (**b**), WT PNS (**c**) and *Ifnar1* KO PNS (**d**) infected with a multiplicity of infection (MOI) of 3.0 for 72 h. Bar: 100 μm. **e, f,** Graphs of total (*grey bars*) and infected (*black bars*) cell densities in mock infected and ZIKV infected CNS cultures at MOI and hours post infection (hpi) indicated. The percentage of infected cells is indicated above each bar. Compared to wild type cultures, more cells are infected in the absence of a type I interferon response. **g, h** Graphs of total and infected cell densities in PNS cultures. Note the different scale on the lower Y axis. Infection rates are very low even in the absence of a type I interferon response. There is a proportion of cells that are false positives, as indicated by black bars in the mock infected cultures. Bars represent mean ± SD; *n* = 3 for all except ‘WT CNS’ and ‘WT PNS mock’ where *n* = 2

To test if ZIKV is type I interferon dependent in this system, we infected WT and *Ifnar1* KO cultures DIV 28, after myelin is established. We treated cultures for 1 h with ZIKV MOI 0.3 or ZIKV MOI 3.0, and fixed the cultures after 24 or 72 h, respectively; the latter was done to increase the opportunity for productive infection. Mock-infected controls were treated with vehicle only. In CNS cultures, markedly more cells were infected in the absence of interferon alpha/beta receptor subunit 1 (IFNAR1), particularly at 72 hpi (compare Fig. 1a with 1b and 1e with 1f). In PNS cultures, the percentage of infected cells was barely above the level of ‘false-positives’ in mock-infected controls, even in the absence of IFNAR1 (Fig. 1c–h). Thus, this Brazilian ZIKV strain is inhibited by type I interferon responses in CNS neural cell cultures, as in other cell types [17], and CNS cells are markedly more susceptible to infection than PNS cells.

### ZIKV infects all major CNS neural cell types

To determine which CNS neural cell types are most susceptible to ZIKV infection, we examined cell-type specific infection at 24 hpi (ZIKV MOI 0.3). In WT cultures, we found evidence for ZIKV in all major cell types except neurones, however the proportion of ZIKV +ve cells, being only 2.4% of all cells (*n* = 2; Fig. 1e), was barely above background levels observed in mock-infected cultures. Consequently, we did not pursue this analysis.

**Fig. 2:**
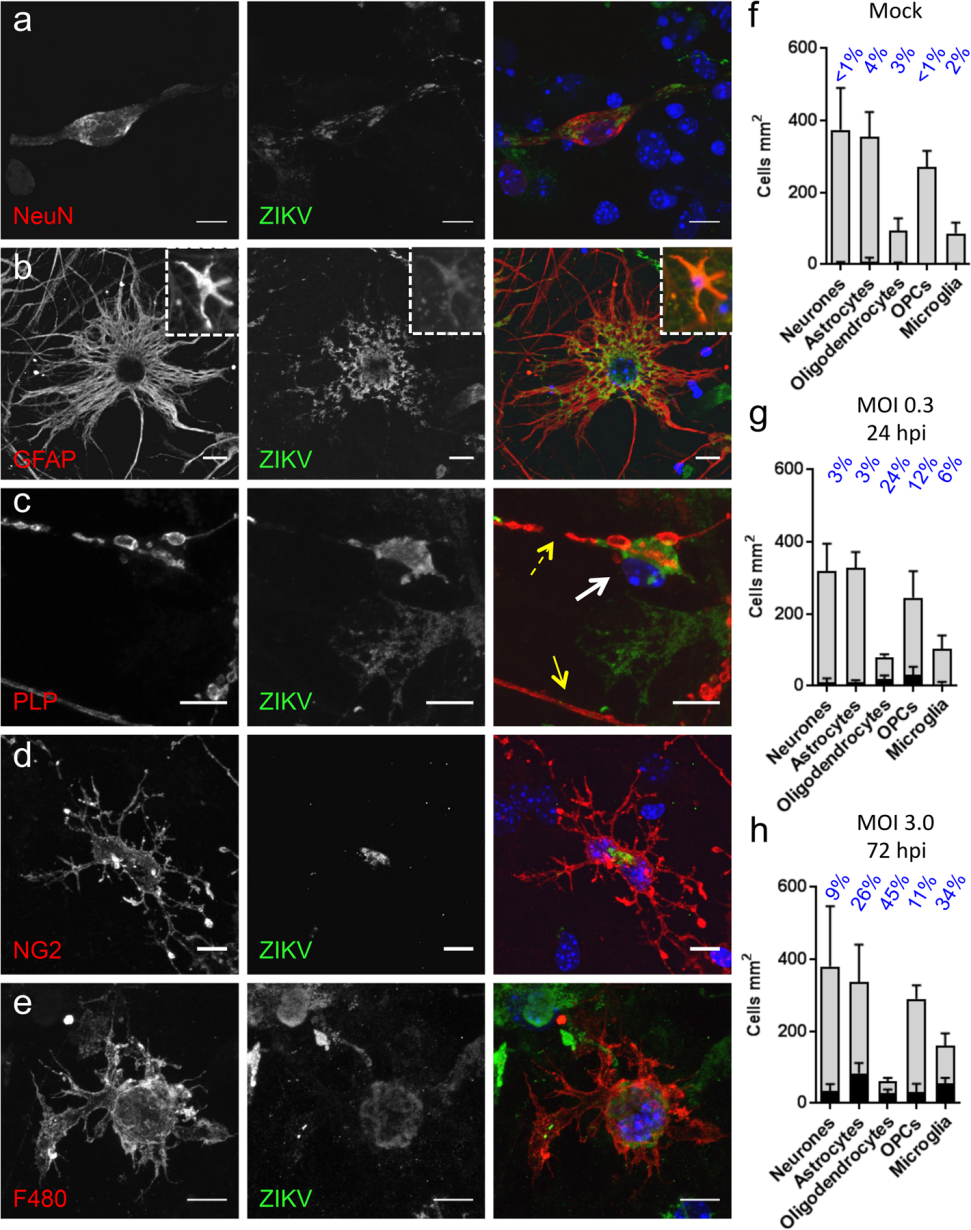
ZIKV infects all principal CNS cell types. **a-e** Micrographs of infected neural cell types from *Ifnar1* KO cultures at 24 or 72 hpi with ZIKV. **a** NeuN-labelled neurone (**b**) GFAP-labelled astrocyte; inset shows an example of an infected cell in which the glial fibrils appear condensed, ​(**c**) PLP/DM20-labelled oligodendrocyte (*white arrow*) with a fragmented myelin sheath (*broken yellow arrow*), below which there is normal-appearing myelin sheath (*solid yellow arrow*), (**d**) NG2-labelled OPCs and (**e**) F480-labelled microglial cell, all containing detectible levels of ZIKV. Bars: 10 μm. **f-h** Graphs showing densities of total (*grey*) and infected (*black*) cells in *Ifnar1* knockout mouse cultures, treated with vehicle (**f**; mock), ZIKV at MOI 0.3 24 hpi (**g**) or ZIKV at MOI 3.0 72 hpi (**h**).​ In mock infected cultures a small number of cells appeared positive for ZIKV and represent false positives. Bars represent mean ± SD; *n* = 3 for all except ‘microglia MOI 0.3 24 hpi’ where *n* = 2

In *Ifnar1* KO cultures, ZIKV was observed to varying extents in all major cell types with the possible exception of neurones which occasionally appeared positive even in mock-infected cultures (Fig. 2a-e illustrates infected cells following ZIKV infection at MOI 0.3 or 3.0 for 24 or 72 hpi, respectively). Overall, the densities of ZIKV +ve *Ifnar1* KO cells were, in decreasing order: oligodendrocyte precursor cells (OPCs), mature oligodendrocytes, astrocytes, neurones and microglia. In these CNS cultures, astrocytes, OPCs and neurones are each present at approximately three times the density of microglia or mature (PLP/DM20 + ve) oligodendrocytes (Fig. 2f). Taking their relative abundance into account, the proportion of each cell type that is ZIKV +ve is, in decreasing order: mature oligodendrocytes (24%), OPCs (12%), microglia (6%), astrocytes (3%) and neurones (3%) (Fig. 2g). We cannot exclude the possibility that the ZIKV +ve staining in microglia is due to phagocytosis of infected material, with or without subsequent replication, rather than direct infection.

As stated above, the titre of ZIKV in the CNS is unknown and will vary spatially and temporally within and between individuals therefore we raised the MOI to 3.0 and the post-infection time to 72 h, as described above. In WT cultures, the proportion of infected cells remained low at 3.9% (*n* = 2; Fig. 1e), but a small proportion of all major cell types, except neurones, were ZIKV +ve (data not shown). In *Ifnar1* KO mouse cultures, all major cell types including neurones harboured detectable levels of virus at this time point (Fig. 2h). Compared to 24 hpi MOI 0.3, there was a change in susceptibility to infection, with the densities of infected cells being, in decreasing order: astrocytes, microglia, neurones, OPCs and mature oligodendrocytes. The relative density of each cell type was not markedly different from that in mock-infected controls (compare grey bars in Fig. 2f and h), suggesting that the composition of the cultures did not change with infection. Taking their relative abundance into account, the proportion of each cell type infected was, in decreasing order: mature oligodendrocytes (45%), microglia (34%), astrocytes (26%), OPCs (11%) and neurones (9%) (Fig. 2h). Most notably, the proportion of infected microglia and astrocytes increased ~5- and ~8-fold, respectively, compared to 24 hpi (MOI 0.3). Despite the longer post-infection time, most infected cells appeared morphologically normal, though a small number of astrocytes were misshapen and the GFAP staining pattern suggested the cytoskeletal filaments were condensed (Fig. 2b insets).

In summary, all major CNS cell types are susceptible to infection in the absence of a type I interferon response, but mature oligodendrocytes are particularly sensitive. Remarkably, compared to the other cell types, there is a disproportionate increase in the percentage of microglia and astrocytes that are infected at 72 hpi (MOI 3.0) relative to 24 hpi (MOI 0.3).

### ZIKV infection is highly pathogenic in CNS cultures

**Fig. 3:**
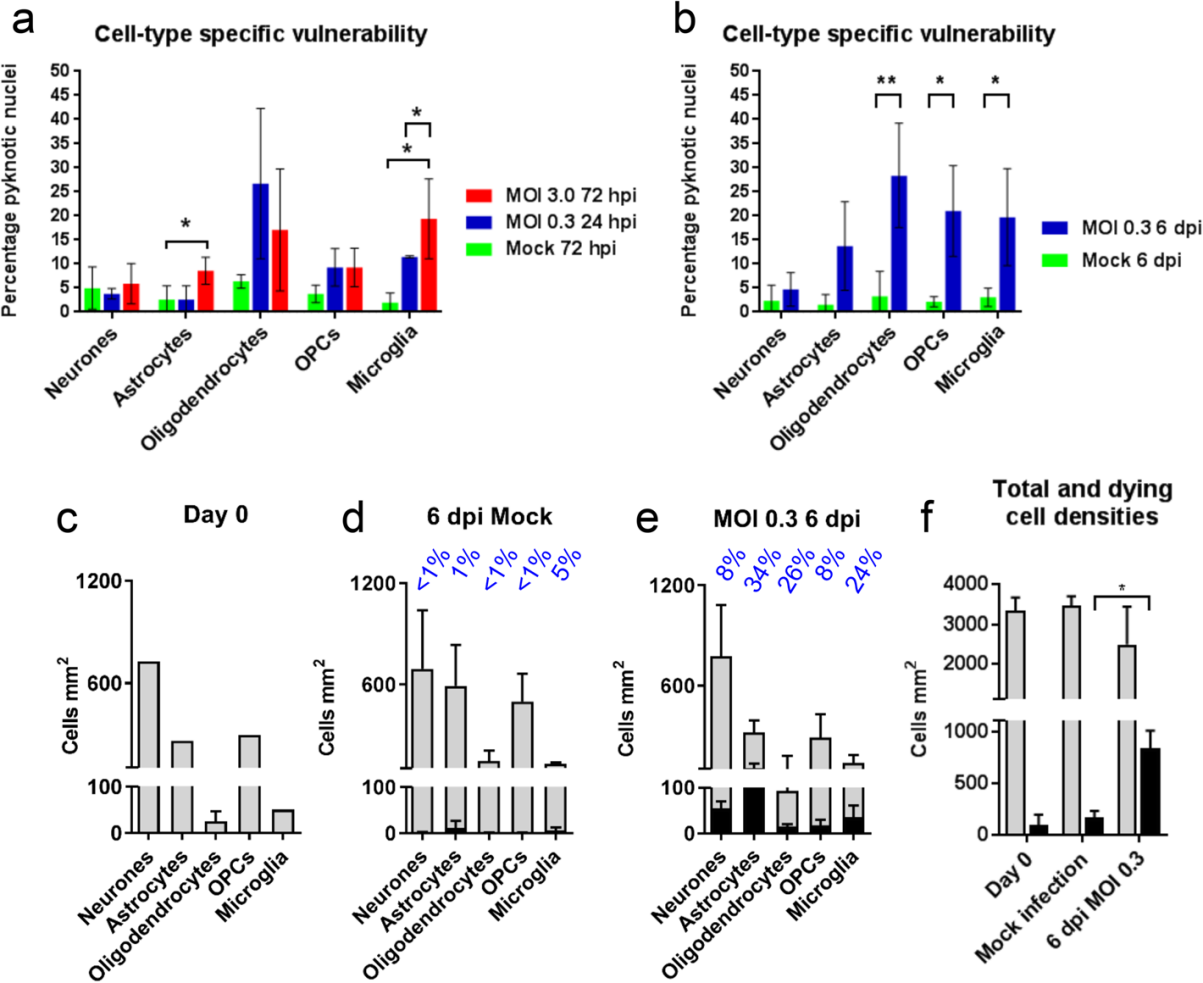
ZIKV infection of CNS cultures causes cell death. **a, b** Graphs of percentage of pyknotic nuclei of each major neural cell type at the post-infection (hpi) time points and MOI indicated. Cell death was assessed on the basis of DAPI +ve nuclear morphology (see M&M). Statistically significant differences relative to mock infected controls (*green*) are indicated by stars. **c-e** Graphs of total densities (*grey bars*) and infected cell densities (*black bars*) of each of the 5 major neural cell types at the day of infection (Day 0; **c**) and at 6 days post infection (dpi) in mock (**d**) and ZIKV (**e**) infected cultures. Percentage infected is indicated above each bar. **f** Graph of healthy nuclei (*grey bars*) and pyknotic nuclei (*black bars*) at day 0 and at 6 dpi in mock infected or ZIKV infected cultures. There is a significant increase in the density of pyknotic nuclei (average 26%) in ZIKV infected cultures compared to mock infected controls (average 5%), although the reduction in the density of healthy nuclei in ZIKV infected cultures does not reach significance. Bars represent mean ± SD; *n* = 3 except DIV 0 counts which are from one experiment only. * = *p* ≤ 0.05; ** = *p* ≤ 0.01

Whilst the CNS cultures and most of their composite cells appeared relatively normal following infection with ZIKV under the conditions described above, we next asked whether ZIKV infection resulted in cell death. In *Ifnar1* KO cultures, 4% (*n* = 3) and 12% (*n* = 3) of DAPI +ve nuclei were pyknotic at 24 hpi and 72 hpi following infection with ZIKV with an MOI of 0.3 and 3.0, respectively, compared to 3% (*n* = 3) in mock-infected controls. We then examined cell type specific vulnerability and found significant increases in dead/dying microglia at both time points and of astrocytes at 72 hpi (Fig. 3a; *n* = 3). There was a trend for PLP/DM20 + ve oligodendrocytes to be susceptible too, but values did not reach significance (Fig. 3a; *n* = 3).

Next we asked if longer-term infection is pathogenic, using *Ifnar1* KO cultures. To address this we treated cultures on DIV 18 (± 2 DIV), when myelin is just beginning to form, with vehicle or ZIKV using an MOI of 0.3 for one hour then assessed cellular composition, myelination and neurite densities at 6 days post infection (dpi).

We observed a significant increase in the proportion of dying oligodendrocytes, OPCs and microglia in ZIKV infected cultures compared to controls (Fig. 3b). In terms of cell-type specific infectivity, ZIKV was detectable in 34% of astrocytes 26% of oligodendrocytes, 24% of microglia, 8% of neurones and 8% of OPCs (Fig. 3e). Despite this, the density of each of the principal neural cell types was not altered markedly in ZIKV infected cultures (Fig. 3e) compared to mock infected controls (Fig. 3d). Nonetheless, infection was accompanied by a slight diminution in total densities of healthy appearing nuclei and a significant increase in total pyknotic nuclei (Fig. 3f).

**Fig. 4:**
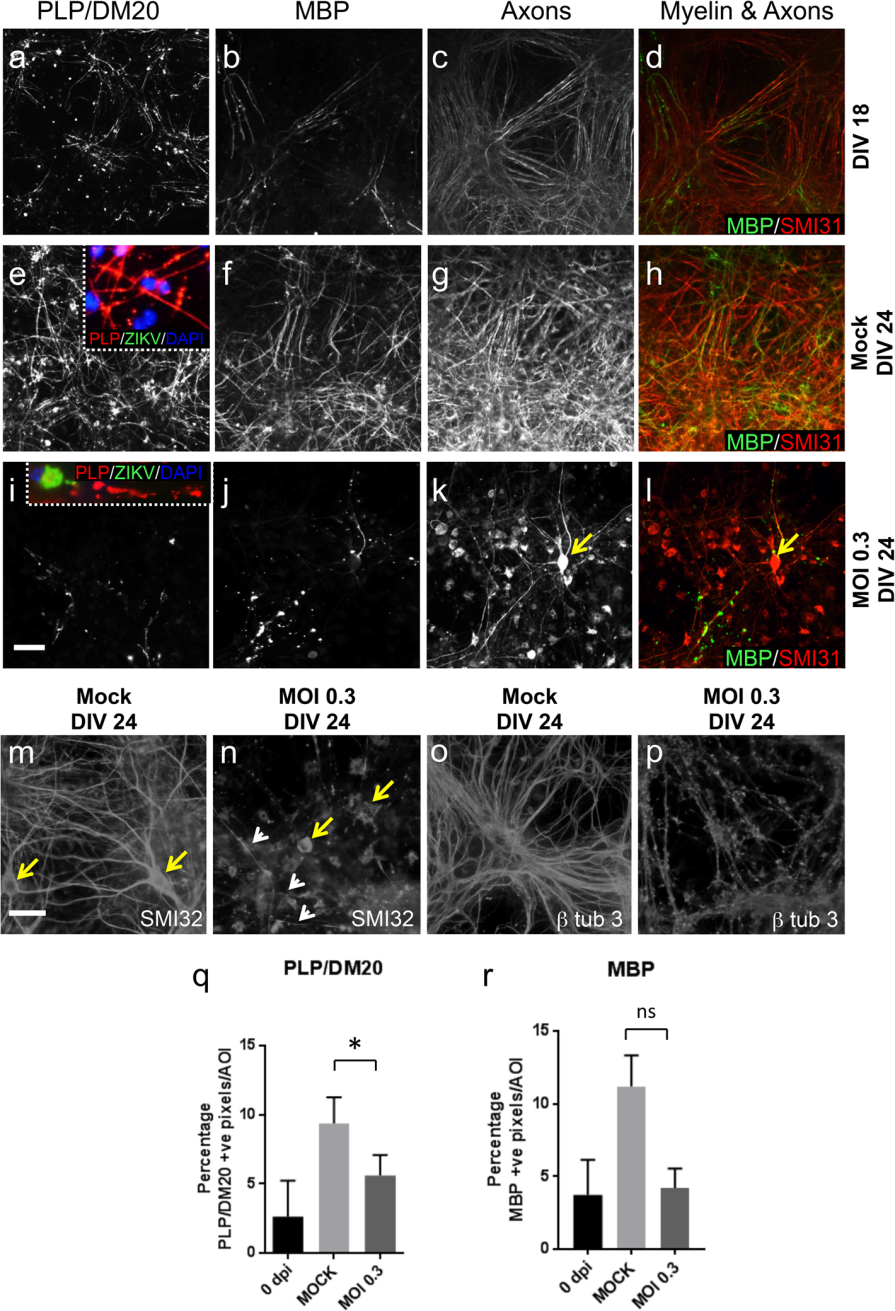
ZIKV infection injures CNS myelin and axons in *Ifnar1* knockout mouse myelinating cultures. Antibody AA3 to PLP/DM20 labels oligodendrocyte cell bodies whilst both AA3 and anti-MBP label myelin-like sheaths at 18 (**a**, **b**, **d**) and 24 days in vitro (DIV) (**e**, **f**, **h**, **i**, **j** and **l**). Antibody SMI31 labels phosphorylated heavy and medium chain neurofilament at 18 (**c**) and 24 DIV (**g**, **k**). **h**, **l** Both myelin and axons are diminished in ZIKV infected cultures compared to mock infected cultures. Most of the remaining myelin in ZIKV infected cultures appears fragmented (inset in i versus inset in e). In contrast to controls, ZIKV infected cultures contain many neuronal cell bodies filled with phosphorylated neurofilament (*yellow arrow* **k, l**). **d, h and l** Deterioration of neurites (axons and dendrites) in ZIKV infected cultures can also be visualized using antibodies to β tubulin 3 (microtubules) and non-phosphorylated neurofilament (SMI32; compare **n** and **p** to **m** and **o).** In n, only a few neurites (*white arrows*) are visible and, whilst normal-appearing neuronal cell bodies are labelled in mock infected cultures (*yellow arrows* in m), those in the infected cultures appear abnormal (*yellow arrows* in n). **q, r** Graphs showing mean percentage of pixels that are PLP/DM20 or MBP positive per area of interest**;** bars represent mean of 3 independent experiments ± SD. Bars: 50 μm,​ * *p* ≤ 0.05

**Fig. 5:**
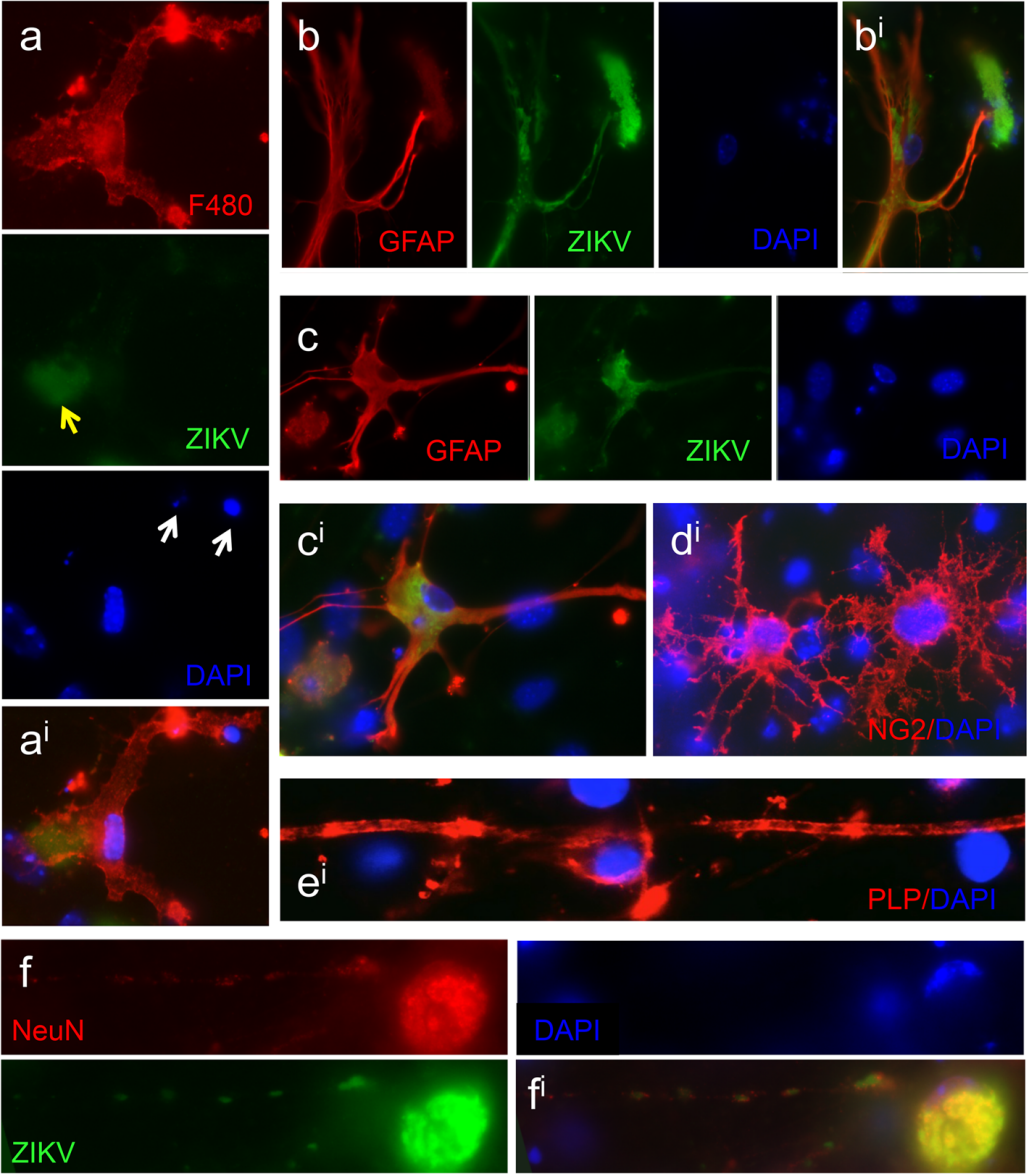
ZIKV infection leads to changes in principal neural cell types in *Ifnar1* knockout cultures. **a** and **a** ^i^ overlay F480-labelled microglia appear to engulf pyknotic nuclei (*white arrows*) and possibly also ZIKV-infected cells (*yellow arrow*). **b**, **b** overlay, **c**, **c** ^i^ overlay Some infected astrocytes appear relatively normal, with filamentous-appearing GFAP staining (**b**), whereas in other cases the filaments appear condensed (**c**). **d** ^i^ The majority of oligodendrocyte precursors were not infected and appeared normal. A proportion of PLP/DM20 positive oligodendrocytes was not infected and retained their myelin sheaths, ﻿**e** ^i^ whereas many of these cells were infected and appear morphologically abnormal with absent myelin sheaths (see Fig. 4). **f**, **f** ^i^ overlay Occasionally neurones were infected and had ZIKV +ve puncta in the cell body and in processes leading from the cell body. In infected neurons and oligodendrocytes, the nucleus often appeared to be displaced to the edge of the soma, as in this case

Strikingly, although we observed no significant decrease in either oligodendroglial or neuronal densities under these conditions (grey bars, Fig. 3d and e), ZIKV infection was associated with a significant decrease in myelin (Fig. 4) as assessed by PLP/DM20 + ve immune reactivity (Fig. 4q). MBP staining was also consistently and markedly reduced, although the reduction did not reach significance due to large variation between different cultures (Fig. 4r). PLP/DM20 and MBP are located in compact myelin, so we examined a 4th independent culture using antibodies to sulphatide, a myelin lipid, and myelin oligodendrocyte glycoprotein (MOG), a protein on the surface of the mature myelin sheath. Both were diminished in ZIKV-infected cultures (Additional file 2: Figure S2). These myelin changes were accompanied by a pronounced qualitative decrease in axons as indicated by antibody staining for phosphorylated neurofilament (compare Fig. 4k and l with g and h), non-phosphorylated neurofilament (compare Fig. 4n with m) and β tubulin 3 (compare Fig. 4p with o). How ZIKV mediates these effects is currently unknown. However, many cells (including oligodendrocytes) had an abnormal morphology (Fig. 4i, inset; Fig. 5) and many neuronal cells bodies were positive for phosphorylated neurofilament heavy and medium chains; an indication of pathology (Fig. 4k and l).

At this time point, some microglia appeared to be in the process of phagocytosing pyknotic nuclei (Fig. 5a) and although many infected astrocytes had normal-appearing GFAP staining (Fig. 5b), filaments appeared condensed in many cells (Fig. 5c). The majority of OPCs were not infected and appeared normal (Fig. 5d) and occasional myelinating oligodendrocytes retained long stretches of smooth myelin (Fig. 5e). ZIKA +ve puncta could be observed in some neuronal cell bodies and in processes (likely dendrites and axons) extending from the soma (Fig. 5f).

In summary, mature oligodendrocytes and axons, but not neuronal cells bodies (as determined with antibody to NeuN) are numerically attenuated following long-term (6 day) infection of CNS cells with ZIKV, and myelin pathology is likely the secondary response to one or other, or both of these events.

### PNS cells are markedly less susceptible to ZIKV infection than CNS cells

**Fig. 6:**
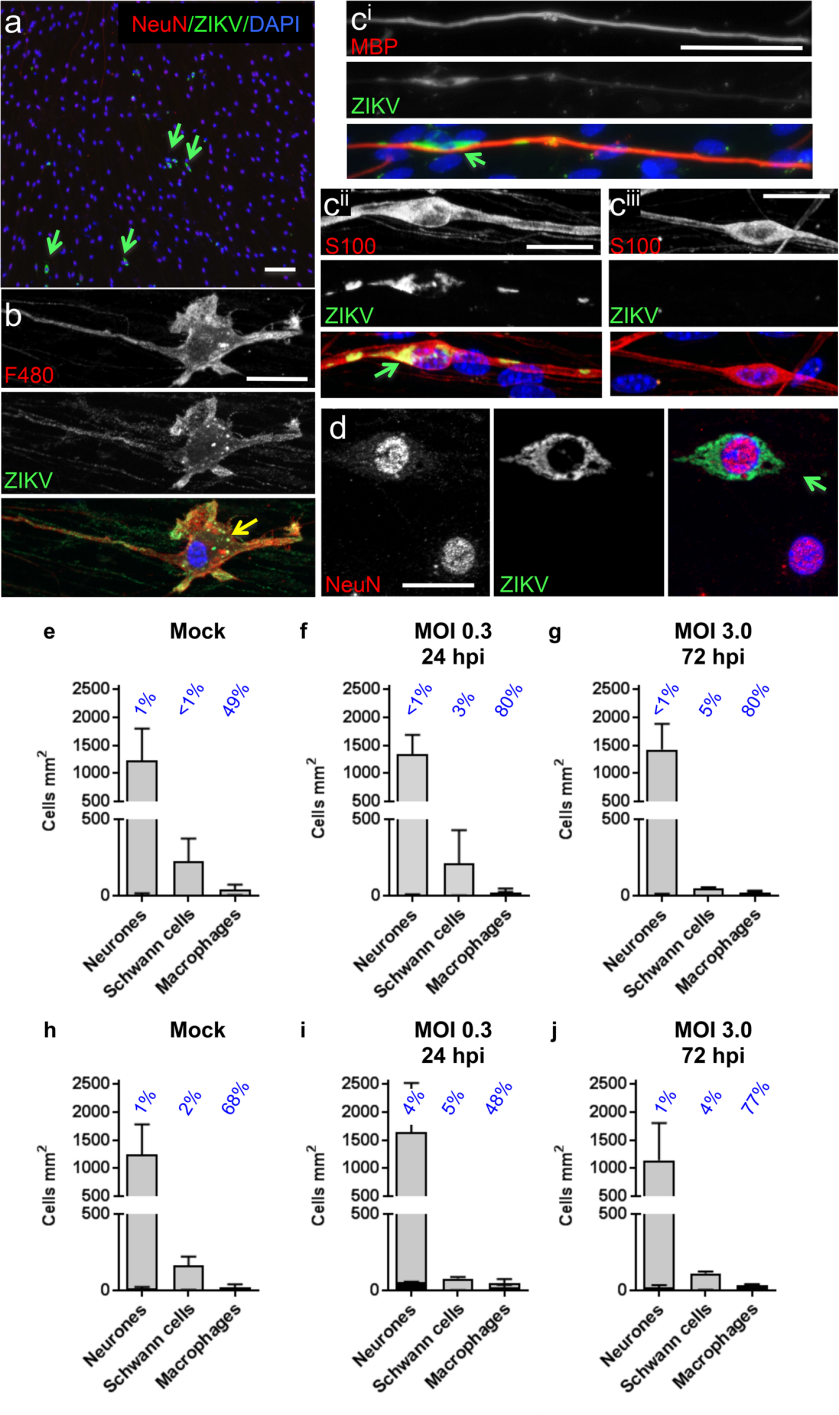
Rarely, DRG neurones and Schwann cells are positive for ZIKV in PNS cultures. **a-d** Micrographs of *Ifnar1* knockout (KO) PNS cultures infected with an MOI of 3.0 for 72 hpi. **a** Overview of a NeuN and ZIKV-labelled culture to illustrate the low overall level of neuronal infection (*green arrows* indicate rare infected cells). Bar: 100 μm. **b** F480 positive macrophage in which green lipofuscin puncta can be mistaken for ZIKV immunostaining as indicated by the level of false ZIKV-positive macrophages observed in mock infected cultures. **c** ZIKV infected MBP-labelled Schwann cell (i) and infected and non-infected S100-labelled Schwann cells (ii and iii, respectively). **d** NeuN-labelled neurones, one of which is infected with ZIKV (*green arrow*). Bars: 20 μm. **e-j** Graphs of cell-type specific densities (*grey bars*) and infected cell densities (*black bars*) in wild type (WT) (**e-g**) and *Ifnar 1* KO cultures (**h-j**). Many macrophages appeared positive for ZIKV in mock-infected cultures, suggesting that many of the macrophages quantified in (**f, g, i** and **j**) represent false positives. *Bars* represent mean ± SD; *n* = 3 for all graphs except mock infected wild type where *n* = 2

As ZIKV infection can cause GBS, we next examined the PNS cultures described briefly above, in more detail. Occasional neurones, Schwann cells and macrophages (as assessed by positive staining for NeuN, S100 and F480 respectively) appeared ZIKV +ve at 72 hpi following infection with ZIKV at MOI 3.0 (Fig. 6a-d), however the overall levels of infection were barely above background levels, even in *Ifnar1* KO cultures. Background immune reactivity was particularly prominent in macrophages, most likely due to the presence of autofluorescent lipofuscin positive granules (Fig. 6b). Nonetheless a few Schwann cells and DRG neurones were considered to be definitively positive for ZIKV (Fig. 6a, c and d). This low level of infection was not due to the presence of inhibitory factors in the culture medium as this media had no effect on the ability of ZIKV to infect A549 cells (data not presented). Even at 12 dpi ZIKV (MOI 0.3), cultures appeared healthy with no overt signs of axonal or myelin pathology or cell death (Additional file 2: Figure S2i).

In summary, our data suggest the PNS is relatively resistant to infection, at least with this ZIKV strain in this ex vivo system.

## Discussion

Understanding the tropism of ZIKV is key to understanding the pathogenesis of ZIKV infection. Here we demonstrate that a Brazilian strain of ZIKV isolated from a patient with no reported neurological symptoms [17], infects and injures CNS cells in murine myelinating cultures. Our data demonstrate that oligodendrocytes are particularly susceptible to infection, although all major CNS neural cells were targeted to varying degrees. Importantly, white matter components -axons and myelinating oligodendrocytes- were particularly vulnerable to ZIKV-induced injury. In contrast, the virus rarely infected PNS cells from the same mouse embryos. Although it would be naïve to discount effects due to viral strain and host species-specific effects, these observations shed new light on the pathobiology of ZIKV-associated neurological syndromes. First, as the cultures model late foetal and early postnatal stages of development in the human nervous system they are valuable for investigating the pathogenesis of neurological symptoms other than microcephaly, which is thought to result predominantly from infection in the 1st trimester [23, 86]. Second, the vulnerability of myelinating oligodendrocytes might explain the extensive white matter pathology associated with congenital ZIKV syndrome. Finally, the inability of ZIKV to productively infect PNS cultures would suggest Zika-GBS is unlikely to be due to direct infection of the PNS, it being more likely Zika-GBS is due to para- or post-infectious autoimmunity, which is the typically recognised pathogenesis pathway for other infection-related forms of GBS.

### ZIKV infection injures white matter structures

Many studies on the pathogenesis of ZIKV have focussed on the proliferation, survival and differentiation of CNS neural precursors; the use of iPS derived human cells conferring species-specific relevance, in some studies [4, 15, 27, 28, 41, 46, 49, 64, 68, 70, 91, 99]. However, it is not currently possible, using human cells, to generate in vitro models of the relatively more mature nervous systems, comprising differentiated neurones and glia and myelinated nerve fibres. Here we took advantage of murine model systems that mimic these aspects, enabling us to identify CNS myelinating oligodendrocytes and axons as particularly vulnerable to ZIKV-induced injury.

Notably, axonal changes occurred in the absence of overt infection or death of neurones. This suggests axons could be injured by soluble or contact-dependent factors in their environment. Previously it has been shown that ZIKV-infected neural crest cells produce cytokines at levels that kill or cause aberrant differentiation of neural progenitors [4]. To date however, the only soluble factor that has been demonstrated unequivocally to injure axons in vivo is nitric oxide [77] and it will be important in future to identify potentially relevant factors in these cell cultures. We and others have previously provided evidence that axonal transport deficits, likely reflecting an energy insufficiency or signalling deficit, can lead to axonal degeneration [19, 20, 39, 57]. In myelinating CNS cultures, cells are suffused in high levels of glucose (25 mM) and pyruvate (1 mM) so the substrates for ATP synthesis are unlikely to be limiting. Nonetheless, if axonal ATP synthesis is attenuated or axonal Ca^2+^ levels are perturbed, for example through mitochondrial injury [13], this could, in principle, account for axonal demise. Future studies will provide mechanistic insight.

The reduction in the amount of myelin at 6 dpi was marked. In this paradigm, cultures were infected at a time when myelin is just forming (~ DIV 18) and the paucity of myelin at 6 dpi could reflect failure of myelin to form normally (dysmyelination) or loss of previously formed myelin (demyelination). The myelin that remained often appeared fragmented (Fig. 4) and many infected PLP/DM20 positive oligodendrocyte cell bodies lacked myelin sheaths. These observations tend to exclude the possibility that myelination is simply arrested. Rather, myelin changes probably represent a combination of the arrest of myelination and the degradation of previously established sheaths. Whether the latter represents a (i) primary pathology, (ii) secondary consequence of oligodendroglial cell death, (iii) secondary consequence of axonal loss or (iv) a combination thereof, remains to be determined.

Importantly, brain white matter is severely compromised in human newborns with congenital ZIKV syndrome caused by infection in the first trimester [12]. Chimelli et al. reported an almost complete lack of myelin and oligodendroglia (Olig 2 positive cells) in hemispheric white matter, and only rare myelinated fibres in the internal capsule and cerebellum in these babies. Axonal changes including spheroids (swellings) and Wallerian degeneration were observed in the brainstem and deep grey nuclei. In contrast, with the exception of the corticospinal tracts, spinal cord white matter appeared relatively normal. Our in vitro data suggest that reasons other than susceptibility to injury explain why spinal cord myelin was preserved in these cases; our CNS cultures are derived from spinal cord.

Spinal cord motor nerve loss has also been observed in newborns with congenital ZIKV syndrome [12, 51]. In contrast, in our spinal-cord derived cultures, neuronal cell death was unremarkable. In these dissociated cell cultures, region specific cell fate determinants are absent or mislocalised and ‘motor neurones’ do not innervate muscle. As a consequence, these cells are unlikely to express the full range of cell-type specific gene products produced in vivo, and this could potentially explain the different observations; although the short-term nature of the cultures is likely also a factor. It will be interesting to ascertain if the small subset of neurones that were infected in our cultures represent a motor neurone population.

In congenital ZIKV cases examined by Chemelli et al., DRGs, dorsal roots and the spinal cord dorsal column appeared normal [12] (the dorsal roots and dorsal columns carry the peripherally and centrally projecting fibres of DRG neurones). The study’s authors speculated the sensory system’s DRG neurones might be less susceptible to ZIKV infection than CNS cells due to their neural crest origin, as is the case for poliomyelitis and WNV encephalitis. Schwann cells are also of neural crest origin, and as demonstrated in the current study, are also much less susceptible to ZIKV than their CNS counterparts, which arise directly from the neural tube.

### All major neural cell types are susceptible to infection by ZIKV

We observed ZIKV envelope protein by immunocytochemistry in all major CNS and PNS neural cell types, however there were marked differences in the susceptibility to infection. Few PNS cells were infected, whilst, in CNS cultures, oligodendrocytes were most susceptible and neurones least susceptible. Astrocytes and microglia displayed a dichotomous infectivity, being only slightly susceptible after short post infection times but markedly more vulnerable, relative to other cells, after long post infection times. Potentially, this could be due to uptake of infectious debris by these cells (for example, debris containing infectious viral RNA or virions). However, in an animal model infected on the day of birth, astrocytes were the first cell to be targeted, being ZIKV +ve at 4 days post infection [91], and the relevance of our observation remains to be ascertained.

### ZIKV infection is enhanced in absence of a type I interferon response

The difference in susceptibility to ZIKV infection between WT and *Ifnar1* KO CNS cultures was marked, as anticipated from previous work [17]. The type I interferon receptor is composed of two polypeptide chains, IFNAR1 and IFNAR2, and binds all type I interferons; the archetypal antiviral cytokines. In the CNS, astrocytes and microglia are thought to be the main source of type I interferons though there is some evidence that oligodendrocytes and neurones might also produce them [65]. Given the low level of infection by ZIKV in both WT and *Ifnar1* KO PNS cultures, it was not possible to ascertain whether type I interferons play a role there too. The expression of interferon receptors in the PNS requires clarification.

### Animal and cell culture models of flavivirus infection

Animal models and animal-derived cells are widely used for neuropathogenesis studies with flaviviruses. Among related neurotropic viruses, JEV has been reported to preferentially infect developing neurones in primary foetal rat brain culture [38] and secondary glial activation is involved in neuronal death [10]; however others have shown that astrocytes and microglia are also susceptible to infection [8, 98]. In mouse models, infection of glial cells and neurones has been described and the effects of type I interferons were studied in terms of the complex interplay between virus detection and subsequent responses [3, 9, 11, 24, 30, 35, 37, 44, 59, 61, 62, 76]. It has been speculated that microglia might act as a reservoir for JEV [84]. Whilst our data suggest ZIKV causes demyelination by direct infection, JEV-associated demyelination has been linked to auto immunity [89]. Some of these key observations have been reproduced in a primate model [60] and human microglia and neurones are also a target for JEV [36, 74, 85]. In the case of WNV, similar studies on tropism and host responses have been conducted [69, 81, 93]. Interestingly, astrocytes have been proposed as persistently infected reservoirs [16] whilst also mediating protective responses and suppression of virus (including WNV and ZIKV) replication [44]. Similar tropism and microglial activation have been described in primate models of WNV [34, 48], as well as in human cells where infection of neurones and glial cells as well as host/inflammatory responses to infection were investigated [7, 92].

## Conclusions

Few data are available for ZIKV infection of the developing CNS or PNS, and our study sheds light on these processes. All cells of the CNS are susceptible to a degree. Recent studies on ZIKV tropism in human brain cell cultures suggest similar tropism (astrocytes, oligodendrocyte precursor cells, microglia and to a lesser extent neurones) [46, 70], demonstrating the relevance of our model. The protein AXL mediates entry into human microglia and astrocytes and modulates innate immune responses [50], confirming previous studies suggesting this host factor is important for ZIKV infection [45, 63]. However, although *Axl* mRNA is expressed in all major neural cell types in the mouse [94], recent studies suggest that AXL is not important for ZIKV infection in mice [33, 42]. Nonetheless, as our murine cultures mimic infection of the human nervous system, but in a readily quantifiable manner, it will be an important tool for studies of ZIKV pathogenesis.

## Additional files

Additional file 1: Figure S1. Cultured E13 spinal cord cells and DRG explants contain myelinated axons. (**a**, **b**) Wild type and *Ifnar1* knockout mouse spinal cord cultures at 28 DIV, labelled with antibodies to phosphorylated neurofilament (NF) and myelin basic protein (MBP). (**c**, **d**) Wild type and *Ifnar1* knockout mouse DRG explant cultures at 28 DIV, labelled with antibodies to NF and MBP. (TIFF 304742 kb)

Additional file 2: Figure S2. ZIKV-related diminution of CNS myelin can be observed using markers of various myelin compartments. Antibody O4 labels the lipid sulphatide **(a, e)** whilst Z2 labels myelin oligodendrocyte glycoprotein (MOG) (**c, g**); both of which are present on oligodendroglia and their myelin sheaths. **a-d** Representative images of mock infected CNS myelinating cultures at DIV 24 show a dense network of myelinated and non-myelinated axons. **e-h** In contrast, ZIKV infected CNS myelinating cultures have a less dense network of neurites (including axons). Myelin is markedly reduced and appears fragmented (**e, g, h**) and some neuronal cell bodies are filled with phosphorylated heavy and medium chain neurofilament (arrows in **f**). Bar: 50 μm. **(i)** In PNS cultures, even at 12 dpi there were no overt signs of myelin pathology or cell death (TIFF 154449 kb)
